# High-Sensitivity and Temperature-Robust Gas Sensor Based on Magnetically Induced Differential Mode Splitting in InSb Photonic Crystals

**DOI:** 10.3390/s26061914

**Published:** 2026-03-18

**Authors:** Jin Zhang, Leyu Chen, Chenxi Xu, Hai-Feng Zhang

**Affiliations:** 1 College of Electronic Engineering, Tongda College of Nanjing University of Posts and Telecommunications, Yangzhou 225127, China; b23020001@njupt.edu.cn (J.Z.); b23021115@njupt.edu.cn (L.C.); 2 College of Electronic and Optical Engineering & College of Flexible Electronics (Future Technology), Nanjing University of Posts and Telecommunications, Nanjing 210023, China; b24020006@njupt.edu.cn

**Keywords:** magneto-optical photonic crystals, refractive index sensing, differential detection strategy, multi-objective dragonfly algorithm, indium antimonide, temperature robustness

## Abstract

High-precision detection of hazardous gases with low refractive indices ranging from 1.000 to 1.100, specifically including methane, carbon monoxide, and sulfur dioxide, is critical for industrial safety, yet conventional sensors often suffer from limited sensitivity and severe thermal cross-sensitivity. This work presents a Magneto-Optical Differential Photonic Crystals Sensor (MO-DPCS) utilizing indium antimonide (InSb) to address these constraints. Employing the Multi-Objective Dragonfly Algorithm (MODA), the system was inversely optimized to maximize magneto-optical polarization splitting while rigorously maintaining an ultra-high transmission efficiency. Crucially, an angular interrogation architecture operating under oblique incidence was established to maximize the magneto-optical non-reciprocity, where the detection was realized by fixing the terahertz source frequency and monitoring the precise angular displacements of the steep spectral edges. A differential detection technique was employed to utilize the non-reciprocal phase changes wherein Transverse Electric (TE) and Transverse Magnetic (TM) modes display contrasting kinematic characteristics in the presence of an external magnetic field. The findings indicate that with an adjusted magnetic field of 0.033 T, the MO-DPCS attains an exceptional differential sensitivity of 30.8°/RIU, much above the 0.8°/RIU seen in the unmagnetized condition. The differential approach efficiently eliminates common-mode thermal noise, minimizing temperature-induced drift to below 0.35° across a 1 K range. The suggested MO-DPCS offers a robust, self-referencing solution for stable and high-sensitivity gas sensing applications with a detection limit of 4.18 × 10^−4^ RIU.

## 1. Introduction

The precise detection and accurate identification of dangerous gases represent a critical imperative in chemical engineering and environmental safety [1,2]. In particular, common hazardous industrial gases such as methane (CH_4_), carbon monoxide (CO), and nitrogen oxide (NO_x_) exhibit extremely low refractive indices, typically ranging from 1.0003 to 1.0008 at standard atmospheric pressure. The release or accumulation of these flammable, explosive, or toxic substances poses severe threats to both industrial infrastructure and public health. Physically, the variation in gas concentration is inherently linked to minute perturbations in the refractive index of the medium, often on the magnitude of 10^−4^ to 10^−5^ RIU. This necessitates sensing devices with an exceptionally low Limit of Detection (*LOD*) to effectively differentiate target analytes from the background environment. Consequently, optical refractive index sensing has emerged as an indispensable technology in chemical analysis [3,4], biosensing [5,6], new energy monitoring [7,8], and environmental governance [9,10]. Leveraging advantages such as non-contact measurement, rapid response, high precision, and resistance to electromagnetic interference, optical sensors have emerged as the predominant method for detection [11,12,13,14]. A variety of sensing designs have been developed, including total internal reflection techniques [15,16], surface plasmon resonance [17,18], interferometry [19,20], and fiber-optic sensing [21,22,23]. Notwithstanding the compactness and stability of these conventional designs, numerous contemporary systems continue to be hindered by restricted resolution and poor quality factors (*Q*) [24,25]. This constraint necessarily results in inadequate sensitivity and a heightened chance of false negatives or overlooked detection in practical applications [26,27]. With the rapid advancement of the internet of things and intelligent manufacturing [28], the demand for sensors that overcome performance limitations has become critical.

To address these challenges, one-dimensional photonic crystals (1D PCs) [29,30,31,32] have garnered substantial attention as a promising sensing platform due to their ease of integration and cost-effective fabrication processes. Recent advancements have significantly augmented the sensing capabilities of these structures. In 2024, Qi et al. [33] demonstrated angle-insensitive ultra-high-*Q* resonances by integrating bound states in the continuum, developing a hypersensitive environmental refractive index sensor and a temperature sensor with maximum sensitivities of 8.67 × 10^5^ μm/RIU and 2.8 × 10^6^ μm/°C, respectively. While the work achieved an impressive detection limit of approximately 10^−5^ RIU within the gaseous refractive index range, it primarily relied on wavelength interrogation, which inherently necessitates high-resolution spectrometers, thereby increasing the system complexity and cost. In 2025, Li et al. [34] introduced an Ω-shaped sensor utilizing gold nanoparticle/polydimethylsiloxane (AuNP/PDMS) core–shell structures for the concurrent measurement of refractive index and temperature. More recently, Wang et al. [35] reported a reconfigurable terahertz (THz) metamaterial utilizing phase-change materials with joint polarization control, successfully demonstrating multifunctional switching and high relative intensity modulation. While these pioneering works have effectively exploited polarization degrees of freedom to expand device functionality, they predominantly rely on absolute parameter shifts, which can be susceptible to environmental fluctuations. In contrast, the differential detection technique employed in this work advances this polarization control strategy by utilizing the non-reciprocal splitting of TE and TM modes to perform a self-referenced subtraction, thereby fundamentally eliminating common-mode thermal noise and significantly amplifying the sensing resolution. Notwithstanding these advancements in spectral detection, angular interrogation techniques have emerged as a robust alternative for the development of compact and cost-effective sensing systems. By operating at a fixed excitation frequency and monitoring the angular shift of resonance modes, angular sensors obviate the requirement for bulky spectral analyzers. Currently, high-performance angular sensing is predominantly realized through surface plasmon resonance (SPR) or defect-mode PCs. However, most existing angular sensors employ passive measurement schemes that track absolute angular shifts, leaving them susceptible to source fluctuations and environmental noise. Therefore, developing an active angular sensing architecture that incorporates differential detection capabilities is critical for further improving measurement stability and resolution. To enhance sensing capabilities from passive detection to active tunable sensing, semiconductor materials are progressively incorporated into dielectric stacks. Indium antimonide (InSb) is a remarkable candidate for the THz spectrum [36,37]. InSb is characterized by its exceptionally low effective electron mass and elevated carrier mobility, giving rise to a pronounced magneto-optical effect [38]. In the presence of an external magnetic field, the permittivity tensor becomes anisotropic, thereby enabling the non-reciprocal manipulation of light, which is a capability absent in passive dielectrics [39]. To further optimize the structural performance, porous silica (porous SiO_2_) is utilized as the low-refractive-index material [40,41]. Porous SiO_2_ constitutes a mature and technologically proven solution for achieving extremely low refractive indices, which is essential for maximizing the refractive index contrast and enhancing the optical field confinement within the device.

The architecture of high-performance magneto-optical devices presents a complex multiparametric optimization problem. Traditional design approaches often rely on trial-and-error iterations or empirical intuition, which are inherently inefficient and prone to entrapment in local optima. The recent surge in artificial intelligence (AI) research has facilitated the integration of intelligent algorithms with nanophotonics, leading to novel opportunities for device optimization [42,43]. In the realm of optical sensing and perception, meta-heuristic algorithms such as Genetic Algorithm (GA) and Particle Swarm Optimization (PSO) have been extensively applied to solve inverse design problems, enabling the precise tailoring of geometric parameters for enhanced light–matter interaction. For instance, in 2023, Teng et al. [44] successfully leveraged genetic algorithms to significantly augment the performance of surface plasmon resonance sensors in a Kretschmann structure through genetic algorithms, exhibiting anti-crossing behavior and achieving an unprecedented sensitivity of 1364°/RIU. In parallel with these optimization techniques, machine learning models have demonstrated remarkable predictive capabilities in sensor characterization. For instance, a Random Forest Regressor (RFR) was recently employed to accurately predict the relative sensitivity and effective material loss of a THz photonic crystal fiber (PCF) sensor designed for explosive detection, achieving high-precision performance forecasting without computationally expensive iterative simulations [45]. However, traditional algorithms frequently encounter challenges related to premature convergence when navigating the high-dimensional search spaces of complex multilayer structures. To execute the inverse design of the sensor architecture in this study, the Multi-Objective Dragonfly Algorithm (MODA) was employed, which was first proposed by Mirjalili in 2015 [46]. Distinct from standard evolutionary algorithms, MODA is characterized by its unique ability to switch between static (exploitation) and dynamic (exploration) swarming behaviors. Drawing inspiration from the natural hunting and migration mechanisms of dragonflies, the algorithm dynamically modulates the weighting factors for separation, alignment, and cohesion. MODA effectively maintains population diversity to avoid local optima while ensuring rapid convergence towards the Pareto front. Such attributes render MODA particularly advantageous for simultaneously optimizing conflicting objectives, such as maximizing the rectangular coefficient (*RC*) [47] while maintaining fabrication feasibility. Through this computational optimization of layer thicknesses, a resonance mode exhibiting an exceptionally high *RC* is attained. This guarantees a spectral profile characterized by a steep and virtually vertical band edge, establishing the physical basis for high-sensitivity detection that manual tuning cannot readily achieve.

Despite these breakthroughs, a significant limitation remains within current sensing methodologies. Most existing refractive index sensors rely heavily on wavelength interrogation, which necessitates bulky and expensive spectral analyzers, or depend on the absolute angular shift of a single resonance peak, leaving them vulnerable to source fluctuations and environmental noise. To address these restrictions, this work introduces a novel Magneto-Optical Differential Photonic Crystals Sensor (MO-DPCS) based on a precise angular interrogation architecture. Unlike intricate lithographic structures, the approach maintains a streamlined, multilayered arrangement while implementing a differential angular detection strategy [48,49,50]. The sensing mechanism is intrinsically governed by the magneto-optical non-reciprocity of the InSb material. Under an external magnetic field, the gyrotropic permittivity of InSb breaks the time-reversal symmetry, inducing a distinct splitting in the dispersion relations of Transverse Electric (TE) and Transverse Magnetic (TM) modes. Consequently, at a fixed THz frequency, the photonic bandgap edges of the two modes exhibit distinct angular cutoff characteristics. By utilizing the angular difference (Δ*θ*) between the TE and TM modes as the sensing signal, this method not only doubles the interrogation efficiency but also efficiently mitigates common-mode noise such as thermal drift and mechanical vibrations. This greatly improves the resolution and stability of the sensor in complex sensing environments.

## 2. Configuration and Methods

**Figure 1:**
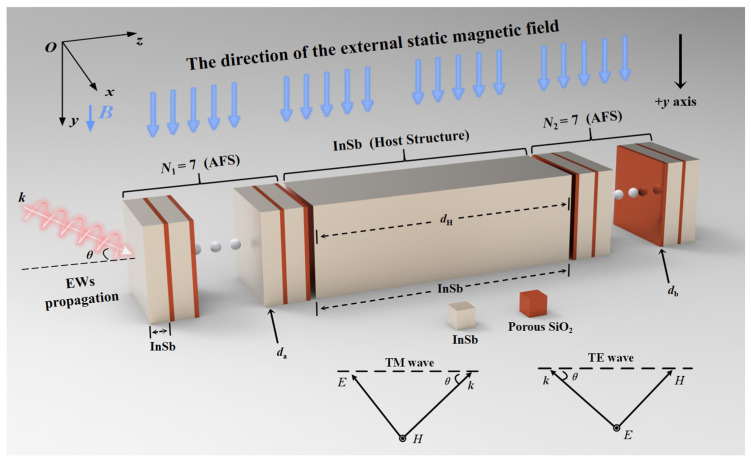
Schematic diagram of the designed sensor MO-DPCS. The host structure is composed of a single layer of InSb material, while the AFSs on both sides are periodic structures consisting of alternating InSb and porous SiO_2_. The blue arrows explicitly indicate the direction of the external static magnetic field *B*, which is applied along the positive +*y*-axis (perpendicular to the plane of incidence) to establish the Voigt configuration.

**Figure 2:**
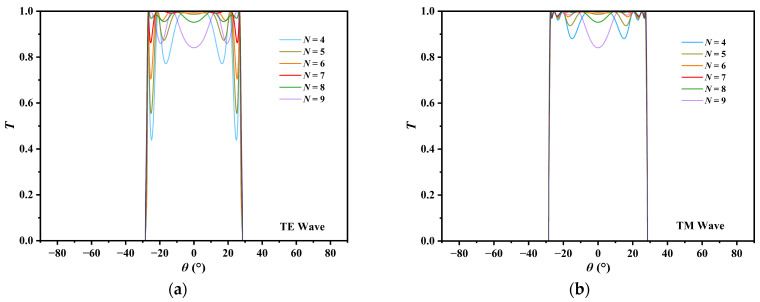
Calculated angular transmission spectra of the MO-DPCS illustrating the impact of the AFS periodicity *N* at an operating frequency of 1.95 THz. (**a**) Transmission spectra for the TE mode with *N* ranging from 4 to 9. (**b**) Transmission spectra for the TM mode under the identical parameter range.

The proposed MO-DPCS is configured as a one-dimensional, multilayered structure tailored specifically for the detection of hazardous gases with low refractive indices, such as CH_4_, CO, and sulfur dioxide (SO_2_). The design focuses on a refractive index spectrum ranging from 1.000 to 1.100. This interval is strategically selected to strictly encompass the refractive indices of vacuum (n = 1.0), atmospheric air (n ≈ 1.0003), and the majority of industrial volatile gases, ensuring comprehensive coverage for gas leakage monitoring. The sensor achieves enhanced sensitivity (S) via a differential sensing methodology. Figure 1 demonstrates that the structure operates as a magneto-optical Fabry–Pérot cavity [51], comprising a central resonant cavity sandwiched between two symmetrical anti-reflection structures (AFSs) [52]. The fundamental host structure comprises a singular layer of InSb with an optimized thickness d_H_ of 434 μm, leveraging the epsilon-near-zero (ENZ) [53,54] property to stimulate a designated angular transmission window (ATW). To reduce signal attenuation caused by thin-film resonance effects at energy transition points, the host structure is interposed between two identical AFSs designed using impedance matching to minimize reflection losses [55,56]. As shown in Figure 2, each AFS consists of 7 periods of alternating layers of InSb, with a thickness of d_a_ = 7.3 μm, and porous SiO_2_ layers, with a thickness of d_b_ = 1.4 μm. This specific periodicity N was rigorously optimized to balance spectral flatness and transmission efficiency, as numerical analysis indicates that lower values result in significant impedance-mismatch ripples within the passband, while higher values induce excessive cavity resonance dips that degrade the flat-top profile. Consequently, the 7-period configuration was identified as the global optimum for securing near-unity transmission. The porous SiO_2_ was chosen for its steady low refractive index and regulated dielectric constant of 1.50. A key aspect of this design is the synchronized modulation of the InSb layers within both the host structure and the AFSs. The device is exposed to an incoming electromagnetic wave (EW) characterized by a wave vector k, which propagates in the xoz-plane (the plane of incidence) at incidence angle *θ*. An external static magnetic field B is oriented along the positive +*y*-axis, maintaining a direction perpendicular to the plane of incidence to establish the Voigt configuration [57]. To practically generate and maintain this uniform magnetic field in industrial environments, a compact current-driven electromagnetic induction module, utilizing a Helmholtz coil configuration, can be integrated into the sensor housing. By passing a regulated DC current through orthogonal conductive windings, a highly directional and spatially linear magnetic field is established along the required axis. Since the optimized field strength of roughly 0.033 T is significantly lower than that required for conventional magneto-optical isolators, this setup avoids the need for bulky superconducting magnets, thereby ensuring that the system remains structurally compact and cost-effective, with negligible increase in complexity. Under this geometric arrangement, the incident light is decomposed into two orthogonal polarization modes. In the TE wave, the electric field vector E oscillates parallel to the +*y*-axis and is thus aligned with B. Conversely, in the TM wave, the magnetic field vector H oscillates parallel to the +*y*-axis, which constrains the electric field vector E to lie within the xoz-plane and remain perpendicular to the direction of B. This configuration generates a significant non-reciprocal magneto-optical effect specifically for the TM mode while maintaining the TE mode as a quasi-static reference, thereby providing the MO-DPCS with enhanced anti-interference capabilities against environmental variations and improved detection resolution by effectively mitigating common-mode noise [58].

The permittivity of InSb can be expressed in tensor form [59]:

(1a) $$\varepsilon_{i}=\left[\begin{array}{ccc} \varepsilon_{x} & 0 & i\varepsilon_{xz}\\ 0 & \varepsilon_{y} & 0\\ -i\varepsilon_{xz} & 0 & \varepsilon_{x} \end{array}\right]$$

(1b) $$\varepsilon_{x}=\varepsilon_{\infty }-\varepsilon_{\infty }\frac{\omega_{p}^{2}(\omega +i\gamma )}{\omega \left[{\left(\omega +i\gamma \right)}^{2}-\omega_{c}^{2}\right]}$$

(1c) $$\varepsilon_{y}=\varepsilon_{\infty }-\varepsilon_{\infty }\frac{\omega_{p}^{2}}{\omega (\omega +i\gamma )}$$

(1d) $$\varepsilon_{xz}=\varepsilon_{\infty }\frac{\omega_{p}^{2}\omega_{c}}{\omega \left[{\left(\omega +i\gamma \right)}^{2}-\omega_{c}^{2}\right]}$$

In this context, *ε*_∞_ denotes the high-frequency dielectric constant of InSb; *γ* signifies the carrier collision frequency [59], which is treated as a temperature-independent constant in this theoretical model. *m* and *e* correspond to the mass and charge of an electron, respectively, and m* equals 0.015 m, indicating the effective mass of the carriers. The theoretical model strictly considers crystalline InSb to leverage its exceptional electronic properties in the THz regime. The high electron mobility and low effective mass inherent to the crystalline state of InSb are fundamentally required to achieve strong magneto-optical non-reciprocity under a relatively low external magnetic field. Compared to amorphous or polycrystalline alternatives, the crystalline lattice effectively minimizes carrier scattering losses, ensuring the ultra-high transmission efficiency and steep spectral edges optimized by MODA. The detailed manufacturing process is provided in Sections S1 and S2 of the Supplementary Materials, the relevant Refs [60,61,62,63]. The emergence of the ENZ property within the investigated THz frequency range is fundamentally rooted in the magneto-plasma resonance response of the semiconductor. To accurately estimate this phenomenon, a Drude tensor model is employed to describe the interaction between the incident electromagnetic waves and the magnetized carriers. When the external magnetic field is applied, the cyclotron motion of the electrons modulates the effective permittivity of the material. At specific frequencies determined by the plasma frequency and the cyclotron frequency, the real part of the diagonal components of the dielectric tensor approaches zero, thereby generating the ENZ state. This near-zero refractive index regime significantly enhances the light–matter interaction within the PC layers, allowing the sensor to exhibit extreme sensitivity to minute variations in the refractive index of the surrounding gas medium. *ω_p_* is the plasma frequency, defined as (*Ne*/*ε*_0_*ε*_∞_m*)^1/2^. Crucially, *ω_p_* serves as a temperature-dependent variable strictly governed by the intrinsic carrier density *N*, which acts as a strong function of the lattice temperature. Here, *ε*_0_ represents vacuum permittivity, while ambient temperature is denoted by *T*_0_. Consequently, the specific temperature-dependent carrier density *N* is specified as [59]

(2) $$N\left({\mathrm{cm}}^{-3}\right)=5.76\times 10^{14}T_{0}^{1.5}\times e^{\left(\frac{-0.26}{2\times 8.625\times 10^{-5}\times T_{0}}\right)}$$

**Figure 3:**
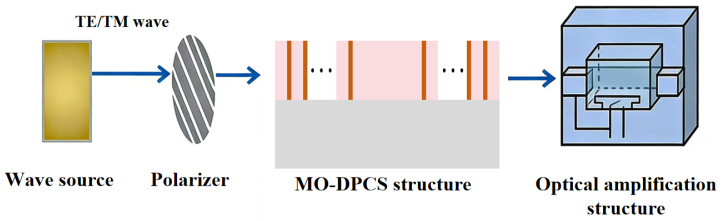
The experimental diagram of MO-DPCS.

To rigorously validate the theoretical predictions under realistic operating conditions, a comprehensive experimental roadmap is proposed based on a linear transmission spectroscopy setup. The measurements are designed to be conducted within a shielded anechoic chamber to eliminate environmental electromagnetic interference. A Vector Network Analyzer (VNA) is employed to drive a transmitting horn antenna positioned on the left side of the sample, launching a collimated electromagnetic wave beam toward the structure. Theoretically, based on the analysis of 1D-PC, the incident wave is a plane wave; the size of the incident plane is not considered, which is equivalent to the incident plane *xoy* being infinite in size. In practical experiments, the spot size of the incident wave should remain consistent with the incident plane. Since the detection mechanism relies on monitoring the precise angular displacements of the steep spectral edges within an angular interrogation architecture, the collimation and coherence of the incident THz beam are critical factors. A high degree of beam collimation with a minimal divergence angle is fundamentally required to prevent the spectral broadening of the band edges. If the incident beam possesses excessive angular divergence, the *RC* of the transmission spectrum will inevitably degrade, thereby reducing the resolution and overall efficiency of the sensing system. To ensure that the performance of the MO-DPCS aligns with the theoretical predictions, the use of a well-collimated continuous-wave THz source is recommended in conjunction with a high-precision rotation stage for accurate angle control. To explicitly verify the polarization splitting capability, a high-extinction-ratio wire-grid polarizer is inserted into the optical path, thereby allowing for the strict isolation of TE and TM modes. The fabricated multilayered structure is mounted on a high-precision motorized rotation stage, enabling continuous angular scanning with millidegree resolution to confirm the steep spectral edges. Furthermore, temperature robustness is assessed by integrating the sample holder with a ceramic heating module and a thermocouple feedback loop. Finally, the transmitted signal is collected by a receiving horn antenna on the right side and fed back to the VNA for *S*-parameter analysis, ensuring that the simulated performance indicators, including angular sensitivity and thermal stability, are corroborated by empirical data. The experimental process of the proposed MO-DPCS is shown in Figure 3. The effective dielectric constant for an incident plane electromagnetic wave in TE mode can be expressed as [59]

(3) $$\varepsilon_{\mathrm{TE}}=\varepsilon_{\infty }\left(1-\frac{\omega_{p}^{2}}{\omega (\omega +i\gamma )}\right)$$

In the Voigt configuration, an external magnetic field *B* is applied perpendicularly to the wave propagation direction, resulting in an anisotropic dielectric response of InSb. In TE mode, the electric field oscillates parallel to *B*, resulting in carrier motion being unaffected by the Lorentz force. Thus, the effective refractive index for the TE mode is isotropic, represented as *n_a_* (TE) = (*ε*_TE_)^1/2^ [64]. Conversely, the TM mode exhibits an electric field orthogonal to *B*, resulting in robust coupling between the EWs and free carriers influenced by the Lorentz force. This interaction is defined by the cyclotron frequency *ω_c_* = *eB*/m* [59], and the effective refractive index in this mode is given by *n_a_* (TM) = [(ε*_x_* − ε*_xz_*)/*ε_x_*]^1/2^ [65].

As a consequence of these distinct dielectric responses, the TE mode retains an isotropic character, whereas the TM mode becomes magneto-optically anisotropic under the influence of the Lorentz force. This divergence in propagation characteristics dictates that within a periodically layered medium, the characteristic transfer matrix of each layer must be formulated independently for the TM and TE waves, which are respectively expressed as [66,67]

(4a) $$M_{n}=\left[\begin{array}{cc} \cos\delta_{n} & -ip_{n}^{-1}\sin\delta_{n}\\ -ip_{n}\sin\delta_{n} & \cos\delta_{n} \end{array}\right]$$

(4b) $$M_{\mathrm{TM}}=\left[\begin{array}{cc} \cos\left(k_{1z}d_{n}\right)+\frac{k_{1x}\varepsilon_{xz}}{k_{1z}\varepsilon_{xx}}\sin\left(k_{1z}d_{n}\right) & -ip_{1}^{-1}\sin\left(k_{1z}d_{n}\right)\\ -ip_{1}\sin\left(k_{1z}d_{n}\right) & \cos\left(k_{1z}d_{n}\right)-\frac{k_{1x}\varepsilon_{xz}}{k_{1z}\varepsilon_{xx}}\sin\left(k_{1z}d_{n}\right) \end{array}\right]$$

(4c) $$M_{\mathrm{TE}}=\left[\begin{array}{cc} \cos\left(k_{2z}d_{n}\right) & -ip_{2}^{-1}\sin\left(k_{2z}d_{n}\right)\\ -ip_{2}\sin\left(k_{2z}d_{n}\right) & \cos\left(k_{2z}d_{n}\right) \end{array}\right]$$

where *k_i_*_z_ = *k_i_* cos *θ_i_* = (*ω*/*c*) *n_i_* cos *θ_i_*, *k*_1*x*_ = *k*_1_ sin *θ_i_*, *p_i_* = (*ε*_0_/*μ*_0_)^1/2^ *n_i_* cos *θ_i_*, and *ε*_0_ = 8.8542 × 10^−12^ represent the vacuum dielectric constant and *μ*_0_ = 4π × 10^−7^ is the permeability of the vacuum [61]. To thoroughly examine the optical transmission characteristics of the proposed MO-DPCS, the equations of Maxwell are resolved utilizing the transfer matrix method (TMM) [15,66,67] under the continuity boundary conditions for tangential electric and magnetic fields, which can be employed to calculate energy transfer between dielectric layers.

## 3. Results

### 3.1. Spectral Characteristics and Algorithm-Driven Optimization of Magneto-Optical Polarization

**Figure 4:**
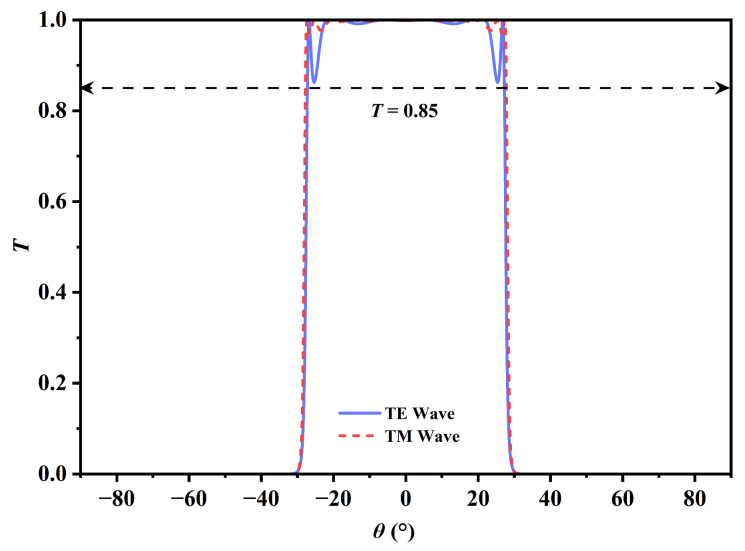
Angular transmission spectra of the MO-DPCS for TE and TM waves calculated at an operating frequency of 1.95 THz under a zero external magnetic field condition.

The optical transmission properties of the proposed MO-DPCS were systematically investigated to validate its capability as a high-performance angular filter. Figure 4 illustrates that the periodic stratification of the structure resulted in a pronounced photonic band gap (PBG) [68,69] attributable to Bragg interference [70]. The MO-DPCS demonstrated an exceptional ATW for both TE and TM waves inside the passband. The transmission efficiency typically surpassed 0.9 across the operational angular range, guaranteeing adequate signal intensity for reliable detection, even in lossy experimental conditions. The steepness of the transmission band edge constituted a critical parameter determining the ultimate detection resolution [47]. A steeper edge indicated a more acute angular transition from the passband to the stopband, thereby reducing the ambiguity in identifying the cutoff angle *θ_cutoff_* and improving the *LOD*. The *RC* was employed as a statistic to quantify the sharpness of the band edge. The *RC* was delineated as [47]

(5) $$RC=\frac{\Delta \theta_{-3\mathrm{dB}}}{\Delta \theta_{-30\mathrm{dB}}}$$

Δ*θ*_−3dB_ and Δ*θ*_−30dB_ represent the angular widths recorded at −3 dB with *T* = 0.5 and at −30 dB with *T* = 0.001, respectively. The rectangular coefficient varied from 0 to 1, with values approaching unity signifying a sharper band edge, thus indicating more angular selectivity and greater measurement precision.

**Figure 5:**
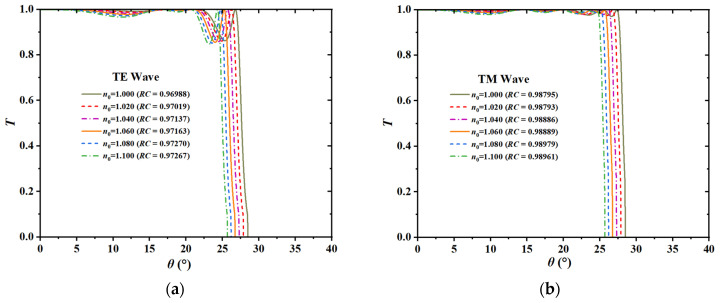
Calculated angular transmission spectra along with the corresponding *RC* values for the MO-DPCS operating at a frequency of 1.95 THz. (**a**) Transmission spectra and *RC* values for the TE mode under varying background refractive indices ranging from 1.000 to 1.100. (**b**) Transmission spectra and *RC* values for the TM mode under the identical refractive index range. The numerical data within the legends indicate that the *RC* remained consistently high, exceeding 0.970 for the TE mode and 0.988 for the TM mode across the entire detection range, thereby demonstrating the superior steepness of the band edge and the structural foundation for high-precision sensing.

Figure 5 illustrates the quantitative assessment of the spectral quality of the proposed sensor. The data reveal that the *RC* values for both TE and TM modes remained consistently above 0.975 across the complete refractive index detection range from 1.000 to 1.100. Simultaneously, the device exhibited exceptional optical throughput, with the peak transmittance of the angular transmission window (ATW) exceeding 99%. This persistently high *RC* indicated a nearly vertical band-edge profile, which was mathematically correlated with a minimized width of the transition region. From a physical perspective, this “flat-top and steep-edge” spectral characteristic offers significant advantages. The high transmission efficiency markedly improves the Signal-to-Noise Ratio (SNR) of the detection system, ensuring that the cutoff point remains distinct even under weak signal conditions, while the high *RC* directly constrains the Full Width at Half Maximum (*FWHM*) of the resonance edge [25]. Since the minimization of the *FWHM* is critical for defining spectral resolution, it serves as the primary mechanism for maximizing the Figure of Merit (*FOM*). Consequently, the *FOM*—where higher values indicate superior quality—serves as the most equitable benchmark for evaluating the comprehensive performance of the sensor in practical applications. Although high-*Q* transmission was crucial, the implementation of differential detection required divergent optical characteristics for TE and TM modes. In the absence of an external magnetic field (*B* = 0 T), InSb functioned as an isotropic material. Under these circumstances, the transmission spectra for TE and TM modes were essentially indistinguishable, with the cutoff angles *θ*_TE_ and *θ*_TM_ approximately coinciding. Thus, any environmental fluctuation could cause simultaneous shifts in both modes, leading to an insignificant differential signal where Δ*θ* ≈ 0. To address this issue, a static magnetic field of 0.033 T was employed in the Voigt configuration. This field effectively disrupted the time-reversal symmetry of the system and induced gyrotropic anisotropy in the InSb layers. Crucially, the Lorentz force exerted a selective influence; it specifically modified the effective refractive index of the TM mode, where the electric field vector *E* was orthogonal to the magnetic field vector *B*. In contrast, the TE mode, with *E* aligned parallel to *B*, remained predominantly unaffected. This magneto-optical polarization splitting generated two distinct signal channels within a single physical structure, creating the necessary bias for the ensuing differential detection technique.

The MODA, a bio-inspired metaheuristic first proposed by Mirjalili in 2015 [46], was employed to rigorously determine the optimal magnetic field strength *B* that maximized the polarization splitting effect while maintaining a high transmission efficiency of the TM mode. MODA represents a sophisticated paradigm in contemporary artificial intelligence research, distinguished by its unique mechanism that mimics the static and dynamic swarming behaviors of dragonflies. In the static phase (exploration), dragonflies form sub-groups to fly over different areas, which allows the algorithm to effectively avoid local optima. Conversely, in the dynamic phase (exploitation), the swarm migrates in a common direction, facilitating rapid convergence toward the global optimum. The core of this algorithm is to calculate the step vectors and position vectors of dragonflies, which are shown as follows, representing their movement directions **Δ*X_i_*** and positions ***X_i_*** [46]. The detailed variable explanations are provided in Table 1 in pseudo-code.

(6a) $$\Delta X_{i+1}=\left(sS_{i}+aA_{i}+cC_{i}+fF_{i}+eE_{i}\right)+w\Delta X_{i}$$

(6b) $$X_{i+1}=X_{i}+\Delta X_{i+1}$$

**Figure 6:**
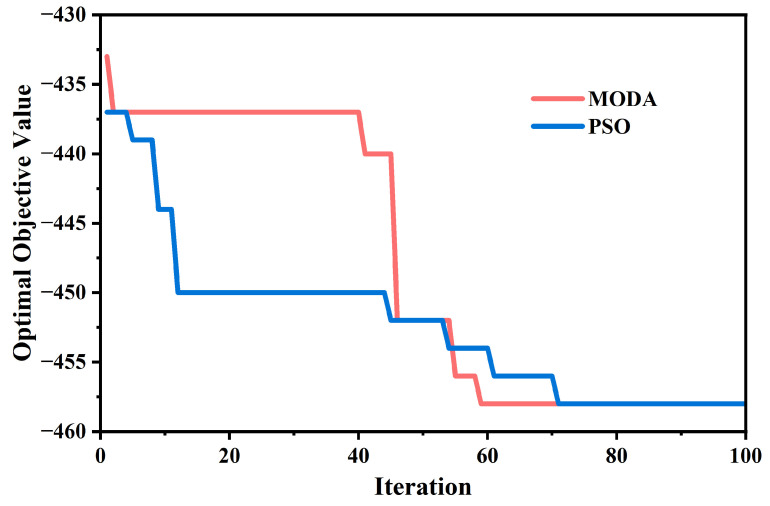
Monte Carlo tolerance analysis of the differential angular response under ±5% layer thickness variations (N = 50). The grey trajectories represent the perturbed structures and the red solid line denotes the ideal design. The statistical analysis yielded a mean sensitivity of 30.55°/RIU with a standard deviation of 5.31°/RIU.

**Figure 7:**
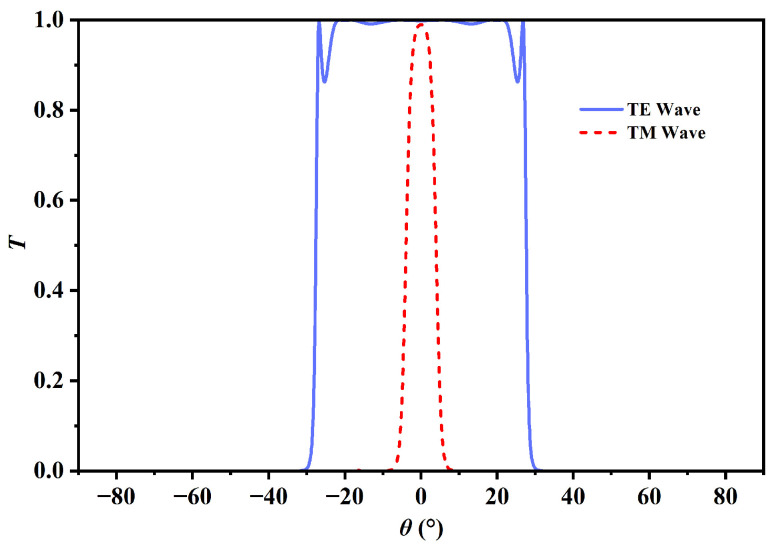
Optimized angular transmission spectra of the MO-DPCS for TE and TM modes calculated at an operating frequency of 1.95 THz under an optimized external magnetic field of 0.032 T obtained via the MODA. The blue and red curves correspond to the TE and TM modes, respectively. The plot visually demonstrates the achieved polarization splitting, thereby validating the effectiveness of the optimization strategy in balancing mode separation with signal integrity.

This exceptional capacity to balance exploration and exploitation makes MODA particularly suitable for addressing intricate, multi-variable engineering challenges with conflicting aims. The efficacy of such AI-driven optimization techniques has been extensively validated in the design of high-performance nanophotonic devices. For instance, Genetic Algorithms have been employed to develop ultra-sensitive SPR sensors, including a generic optimization technique that identified a series of sensors with sensitivity improvements of approximately 100%. Notably, a dual-mode SPR structure coupling Surface Plasmon Polaritons and waveguide modes in germanium dioxide was achieved, exhibiting anti-crossing behavior and an unprecedented sensitivity of 1364°/RIU [44]. Other GA-optimized designs include bimetallic Al/Ag structures sandwiched in hBN (578°/RIU at 633 nm) and hybrid hBN/MoS_2_/hBN structures (676°/RIU at 785 nm). Furthermore, deep learning approaches such as Convolutional Neural Networks (CNNs) have proven superior to traditional methods in the inverse design of Photonic Crystal Waveguides (PCWs). Hybrid frameworks combining the Grey Wolf Optimizer (GWO) with CNNs have also been utilized to optimize complex D-shaped PCF polarization filters, achieving a crosstalk of 965.3 dB and high confinement losses [43]. These cases underscore the powerful potential of intelligent algorithms in pushing the performance boundaries of photonic sensors. As shown in Figure 6, it is acknowledged that similar optimization outcomes can be achieved by other meta-heuristic algorithms, such as GA or PSO. However, MODA is selected in this work to explore its specific efficacy in the context of one-dimensional magnetic PCs, representing a contemporary research direction for solving complex non-linear problems. To ensure the reliability of the optimized parameters, a comparative analysis was performed between MODA and PSO. It was observed that identical convergence toward the global optimum is achieved by both methods, confirming that the optimized configuration is independent of the specific algorithm employed and that the results derived from MODA are robust and consistent. In this study, the optimization focused on a highly refined search space, as the requisite magnetic field adjustments were extremely subtle yet decisive for the resolution of the sensor. The algorithm was assigned the duty of navigating this delicate trade-off to determine a specific magnetic field intensity that maximized the divergence between TE and TM modes while specifically maintaining a high transmission rate. Through rigorous iterative optimization, a magnetic field of 0.032 T was determined to be the global optimum. Figure 7 illustrates the optical response of the structure under optimal conditions, showcasing significant polarization separation while maintaining TM passband transmission above 0.9 (with maximum transmissivity reaching 0.99). The detailed implementation logic and the mathematical foundation of the optimization technique are encapsulated in the pseudo-code provided in Algorithm 1.
**Algorithm 1:** Multi-Objective Dragonfly Algorithm for Magnetic Field Optimization
**Initialize population:****for** *i* = 1 to *N* **do** **|**  Magnetic field range: ***X_i_*** = range(*B*_min_, *B*_max_)       // Magnetic field initialization**end**Archive is empty **Iteration**
*M*:**for** *n* = 1 to *M*_max_ **do** ** |  Update** adaptive parameters: *w*, *s*, *a*, *c*, *f* and *e*         // Update Pareto** |  Update** archive and select food/enemy position      // Minimize objectives** |  for** *i* = 1 to *N* **do**** |   |**  Separation: ***S_i_*** ← -sum(***X_j_***) − ***X_j_***                              // Update positions ** |   |**  Alignment: ***A_i_*** ← mean(**Δ*X_j_***)** |   |**  Cohesion: ***C_i_*** ← mean(***X_j_***) − ***X_i_*** ** |   |**  Food: ***F_i_*** ← Food position − ***X_i_*** ** |   |**  Enemy: ***E_i_*** ← Enemy position + ***X_i_***** |   |  Calculate** step vectors and position vectors** |   |       Δ*X_i+_*_1_** = *s* ×***S_i_*** + *a* ×***A_i_*** + *c* ×***C_i_*** + *f* ×***F_i_*** + *e* ×***E_i_*** + *w* × **Δ*X_i_***** |   |       *X_i+_*_1_** =***X_i_*** *+*
**Δ*X_i+_*_1_**** |   |  Update results**** |  end****end****return** Archive_*X*, Archive_*F*                                           // Output results

To comprehensively execute the inverse design and ensure the seamless integration between the meta-heuristic optimization and TMM calculations, the initialization configuration of MODA was meticulously defined. The optimization framework is multifaceted, encompassing global algorithmic settings, dynamically adaptive swarming coefficients, and strict electrodynamic constraints. Notably, the swarming parameters, such as separation, alignment, and cohesion weights, are non-static and adaptively transition from the exploration phase to the exploitation phase as iterations progress. Furthermore, the objective functions were evaluated under high-resolution angular interrogations and strictly bounded by intrinsic physical properties. The complete architectural parameters and boundary conditions dictating this complex optimization process are systematically detailed in Table 1.

### 3.2. Refractive Index Sensing Response and Sensitivity Analysis

**Figure 8:**
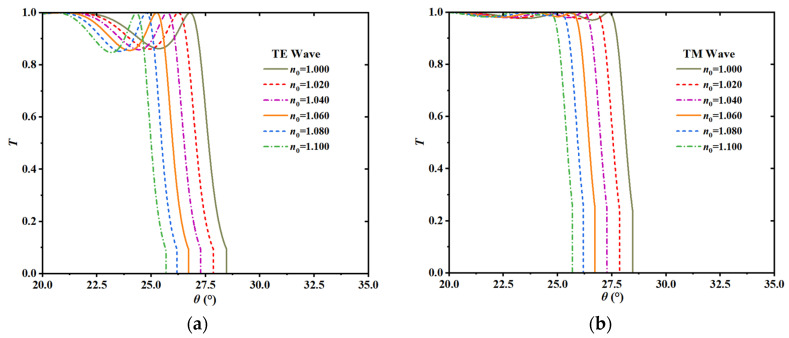
Angular transmission spectra of the sensor calculated at an operating frequency of 1.95 THz in the absence of an external magnetic field, where *B* was 0 T. (**a**) Transmission response for the TE mode as the background refractive index varied from 1.000 to 1.100. (**b**) Transmission response for the TM mode under the identical refractive index range. The observed synchronous shift of both modes towards smaller incident angles signifies the conventional baseline response of the structure prior to magnetic modulation.

**Figure 9:**
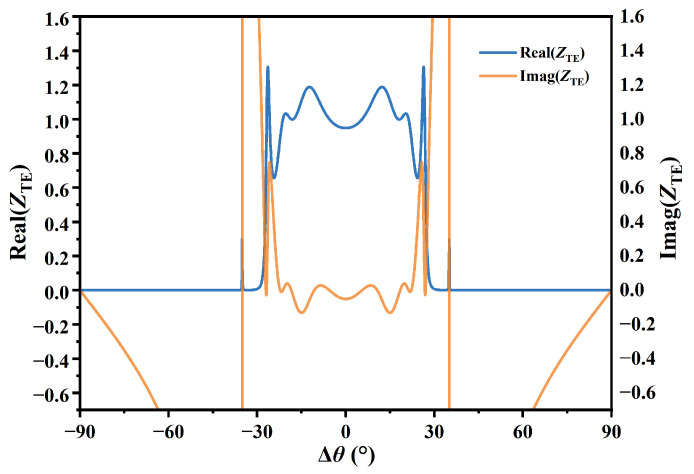
Calculated effective surface impedance of the proposed structure for the TE mode. The blue and orange curves represent the real part (Real (Z_TE_)) and imaginary part (Imag (Z_TE_)) of the normalized impedance, respectively, demonstrating the impedance matching condition at the resonance angle.

The primary sensing mechanism of the proposed MO-DPCS relies on the dependence of the PBG edge on the dielectric surroundings. Variations in the target gas concentration caused fluctuations in the background refractive index *n*_0_, which subsequently modified the effective optical path length within the crystal layers. This alteration in the Bragg resonance condition resulted in a notable angular displacement of the transmission window edge. To clarify the precise effect of magnetic modulation, the response without external magnetic field *B* = 0 T was initially analyzed to establish a baseline. Specifically, Figure 8a and Figure 8b illustrate the transmission spectra for the TE and TM modes, respectively, demonstrating a concurrent trend towards reduced incidence angles as the refractive index increased from 1.000 to 1.100. While this synchronous blue-shift is characteristic of the unmagnetized state, the application of the optimal external magnetic field induced a distinct magneto-optical phenomenon. Under these magnetized conditions, the two orthogonal polarizations demonstrated contrasting shifting tendencies. The cutoff angle of the TE mode progressively decreased, whereas the cutoff angle of the TM mode shifted in the opposite direction and increased. As shown in Figure 9, to unravel the physical origin of this polarization splitting, the effective surface impedance of the structure was theoretically analyzed. It is a fundamental electromagnetic principle that maximal optical transmission through a metastructure occurs only when the effective impedance is perfectly matched to the intrinsic impedance of free space, specifically, when the real part of the normalized impedance (*Z_eff_*/*Z*_0_) approaches unity and the imaginary part approaches zero. Our calculations reveal that for the resonant TE mode, this critical matching condition was strictly satisfied, with the reactance vanishing (Imag (*Z*_TE_) ≈ 0) and the resistance matching free space (Real (*Z*_TE_) ≈ 0), thereby facilitating resonant tunneling. While the TM mode was equally capable of achieving such impedance matching, the gyrotropy induced by the external magnetic field broke the symmetry between the two polarizations. This anisotropy caused the impedance matching conditions for the TE and TM modes to diverge in terms of the operational angle. Consequently, at the specific angle where the TE mode was perfectly matched and transmitting, the TM mode encountered a significant impedance mismatch characterized by a non-zero reactance. This impedance barrier effectively suppressed the TM transmission, confirming that the experimentally observed mode splitting was fundamentally governed by the polarization-dependent impedance modulation of the magneto-optical medium.

**Figure 10:**
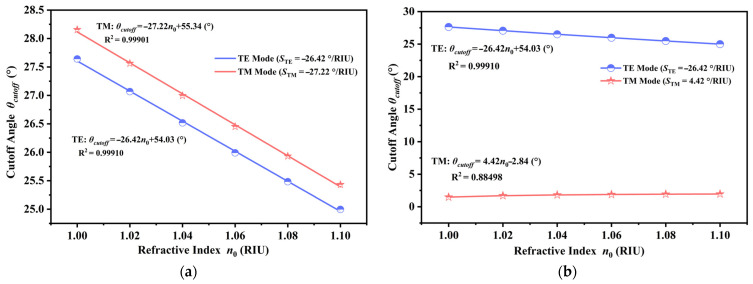
Linear fitting of the angular sensitivity for the proposed sensor under distinct magnetic field configurations. (**a**) Dependence of the cutoff angle on the refractive index in the absence of an external magnetic field where *B* is 0 T, illustrating a synchronous blue-shift for both modes. (**b**) Dependence of the cutoff angle on the refractive index under the optimized magnetic field of 0.032 T. The numerical fitting results in Figure 10b reveal the critical reverse-motion phenomenon, where the TE mode maintained a negative sensitivity of −26.42°/RIU, while the TM mode demonstrated a positive sensitivity of +4.42°/RIU.

The sensitivity, denoted as *S*, was computed for both modes and is fundamentally defined as the slope of the angular shift in relation to the change in refractive index. As depicted in Figure 10a, in the unmagnetized state, both modes exhibited a highly similar synchronous response. The TE mode follows the linear equation *θ_cutoff_* = −26.42 *n*_0_ + 54.03° with a correlation coefficient (R^2^) of 0.99910, yielding a sensitivity *θ_TE_* of −26.42°/RIU. Concurrently, the TM mode is described by *θ_cutoff_* = −27.22 *n*_0_ − 55.34° (R^2^ = 0.99901), resulting in a sensitivity *θ_TM_* of −27.22°/RIU. Upon the application of the optimized external magnetic field, as shown in Figure 10b, the two orthogonal polarizations demonstrate contrasting shifting tendencies. The TE mode, remaining unaffected by the magnetic field, maintained its negative angular shift trend with *θ_TE_* = −26.42°/RIU. Conversely, the TM mode experienced strong Lorentz force coupling, causing its cutoff angle to progressively increase. This transition was mathematically defined by the linear fitting equation *θ_cutoff_* = 4.42 *n*_0_ − 2.84° (R^2^ = 0.88498), which corresponded to a positive sensitivity *θ_TM_* of +4.42°/RIU. While exceptional linearity was maintained by the TE mode, a relatively lower global R^2^ of 0.88498 was exhibited by the TM mode, which was attributed to the non-linear dispersive nature of the magneto-optical effect across the broad detection range. It should be noted that this global fitting is presented to represent the worst-case scenario regarding the trending behavior of the system. To ensure calibration reliability in practical sensing applications, a piecewise linear fitting strategy can be employed, wherein the detection range is segmented into smaller intervals to achieve local R^2^ values exceeding 0.99. Furthermore, the final sensing output was derived from the differential angular shift, which, as evidenced in the subsequent analysis, retained high linearity (R^2^ > 0.99) and resolution, thereby demonstrating that the overall accuracy of the system remained uncompromised. Although the absolute values of the sensitivities varied, the essential discovery was their contrasting signals. This unique differentiation in directional response established the physical basis for the ensuing differential detection technique, which was employed to enhance the overall sensor responsiveness and mitigate common-mode noise.

### 3.3. Differential Detection Strategy and Comprehensive Performance Evaluation

To enhance the sensing capability of the proposed structure, a differential detection scheme was adopted to quantify the sensing signal. Unlike conventional detection methods, where the sensor response relies on a single resonance feature that is susceptible to environmental noise, the response in this system is determined by the absolute difference between the cutoff angles of the two orthogonal polarization modes. The differential angular shift, denoted as Δ*θ_diff_*, represents the separation between the TE and TM cutoff edges. Consequently, the total differential sensitivity *S_diff_* is theoretically defined as the absolute value of the disparity between the individual sensitivities.

(7) $$S_{diff}=\left|S_{\mathrm{TE}}-S_{\mathrm{TM}}\right|$$

**Figure 11:**
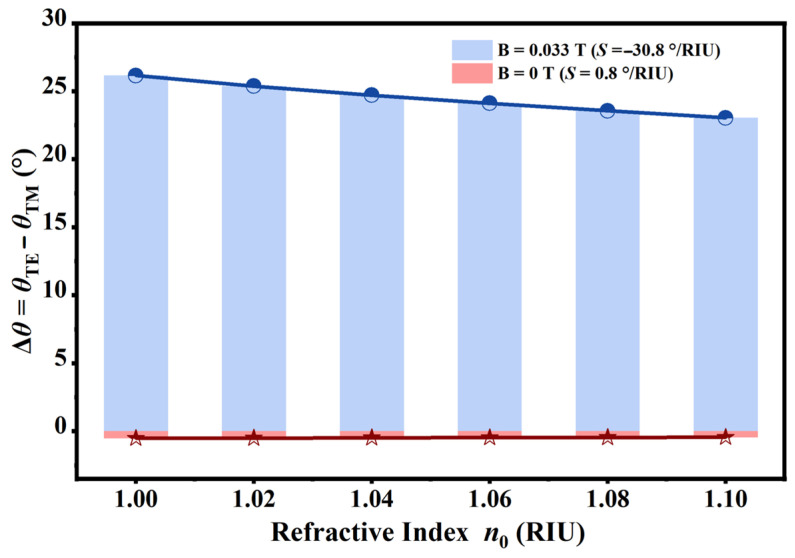
Comparative analysis of the differential angular shift as a function of the refractive index under distinct magnetic field configurations. The response observed under the optimized magnetic field where *B* = 0.033 T exhibited a substantial differential sensitivity magnitude of 30.8°/RIU. Conversely, the results obtained in the absence of an external magnetic field where *B* = 0 T yielded a negligible sensitivity of 0.8°/RIU. This distinct contrast demonstrates the significant sensitivity enhancement achieved through the proposed magneto-optical differential detection strategy.

The effectiveness of this differential method relies significantly on the directional shifting patterns of the orthogonal modes. As illustrated in Figure 11, the differential angular shift exhibited completely distinct behaviors depending on the external magnetic field configuration. In the absence of an external magnetic field (*B* = 0 T), both modes underwent synchronous blue-shifts. Because the algebraic signs of the individual sensitivities *S*_TE_ and *S*_TM_ were congruent, the differential computation inherently resulted in the subtraction of their magnitudes. Quantitative linear fitting verified this limitation, yielding the equation Δ*θ* = −0.8 *n*_0_ − 1.31°, with R^2^ of 0.9935. This corresponded to a negligible differential sensitivity of merely 0.8°/RIU, offering minimal advantage over conventional single-mode detection. Conversely, the application of the external magnetic field (*B* = 0.033T) exploited the unique reverse-motion characteristic, wherein the individual sensitivities exhibited opposing signs. Under this condition, the algebraic subtraction of the negative *S*_TE_ from the positive *S*_TM_ resulted in a constructive superposition of their absolute values. The linear fitting analysis for this magnetized state defined the relationship as Δ*θ* = −30.84 *n*_0_ + 56.87° (R^2^ = 0.9941), which confirmed a markedly enhanced differential sensitivity of 30.84°/RIU. This exceptional sensitivity significantly surpassed the performance attainable in the unmagnetized state, conclusively demonstrating that the magneto-optical effect served as the primary driver for the superior analytical performance of the proposed sensor.

**Figure 12:**
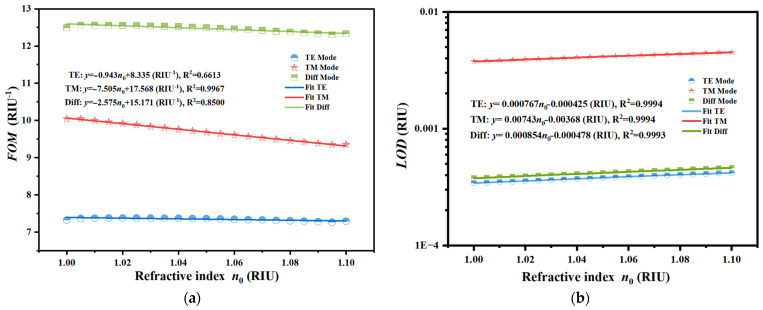
Comprehensive performance evaluation of the MO-DPCS comparing TE, TM, and differential detection mode under the optimized magnetic field. (**a**) Calculated *FOM* as a function of refractive index, where the differential mode exhibited the highest values, confirming its superior spectral quality and sensitivity balance. (**b**) Calculated *LOD* as a function of refractive index using a logarithmic scale, revealing that the differential mode achieved the lowest detection limit of approximately 4.18 × 10^−4^ RIU, demonstrating the enhanced capability in resolving minute refractive index variations.

**Figure 13:**
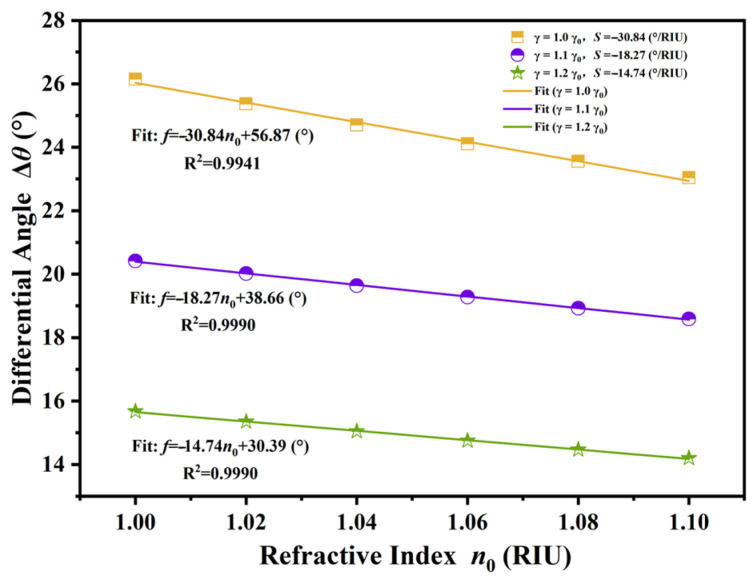
Linear fitting results of the differential angular shift as a function of refractive index under varying collision frequencies (*γ*). As the material loss factor increased from 1.0 *γ_0_* to 1.2 *γ_0_*, the differential sensitivity was observed to decrease from 30.84°/RIU to 14.74°/RIU.

**Figure 14:**
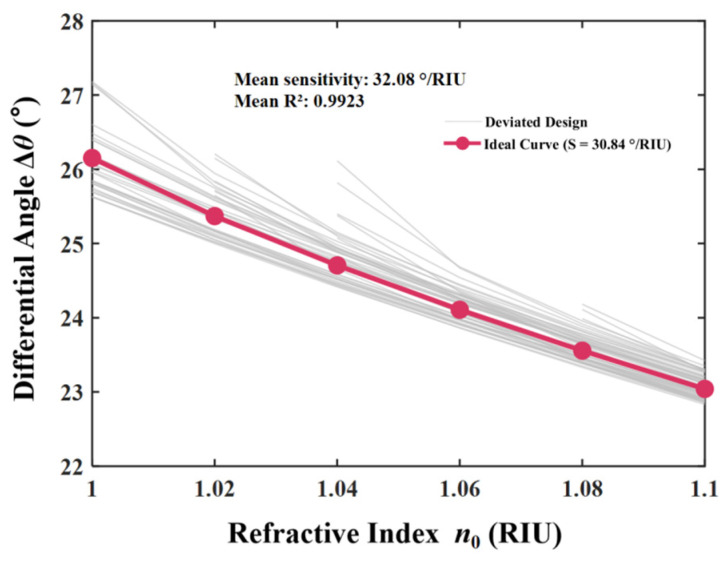
Fabrication tolerance analysis of the proposed MO-DPCS. The gray lines represent the differential angular shift (Δ*θ*) results from multiple independent Monte Carlo simulation cycles, where random thickness variations were introduced to the constituent layers. The red solid line indicates the performance of the ideal curve. The statistical distribution confirmed that while geometric deviations caused minor fluctuations in the response, the linear relationship between the differential signal and the refractive index was predominantly preserved, demonstrating the structural robustness and practical feasibility of the sensor under realistic manufacturing conditions.

A comprehensive evaluation of the realistic resolution limit was conducted by carefully assessing the *FWHM*, *FOM*, and *LOD* of the sensor. As delineated in Section 3.1, the elevated *RC* of the structure guaranteed an almost vertical band edge, which immediately correlated with the reduced *FWHM* of the derivative spectrum. This spectral acuity was essential for performance enhancement. The evaluation was fundamentally grounded in the optimized differential sensitivity, which demonstrated a robust linear response characterized by the equation Δ*θ* = −49.090 *n*_0_ + 75.554°, with a correlation coefficient of R^2^ = 0.9979. The *FOM* was defined as the ratio of sensitivity to *FWHM*. The proposed sensor attained remarkable figures of merit due to this enhanced differential sensitivity combined with the reduced *FWHM*. Figure 12a illustrates that the differential mode displayed the highest *FOM* throughout the full detection range, markedly exceeding the individual TE and TM modes. This superior performance was quantitatively defined by the linear fitting equation *y* = −2.575 *n*_0_ + 15.171 /RIU with R^2^ = 0.8500. Furthermore, the *LOD* was inversely related to the product of sensitivity and the quality factor, mathematically represented as the *FWHM* divided by twenty times the sensitivity. Figure 12b demonstrates that the differential mode achieved the optimal resolution capability. The linear regression for the differential *LOD* was described by *y* = 0.000854 *n*_0_ − 0.000478 RIU, with an exceptional linearity of R^2^ = 0.9993, ultimately reaching a minimal detection limit of roughly 4.18 × 10^−4^ RIU. To practically realize this reported *LOD*, an angular resolution of approximately 0.013° was required by the detection system. This level of precision is experimentally realistic and well within the capabilities of standard commercial high-precision goniometers. Furthermore, to ensure the distinct capture of minute refractive index variations, a geometric optics detection scheme utilizing a reflective lens group can be employed. By functioning as an optical lever, the angular displacement of the beam is significantly magnified, thereby allowing the subtle deflection angles to be resolved with high acuity. This capability is particularly vital for distinguishing gases with similar refractive indices, such as CH_4_, CO, and NO_x_. Although the refractive index values are proximate, distinct angular shifts are induced by the high differential sensitivity of the system. Through the application of the aforementioned geometric optics magnification, these minute deviations are translated into separable readout signals, thereby ensuring that specific gaseous species can be effectively differentiated even within mixed environments. This superior performance metric indicates that the sensor is capable of resolving extremely minute variations in the refractive index, thereby validating the suitability of the proposed device for high-precision biosensing and gas detection applications. As shown in Figure 13, prior to a comparative assessment, the influence of intrinsic material absorption in the THz regime was rigorously addressed. In TMM, these losses were fully quantified by the collision frequency *γ* within the permittivity tensor. While the proposed sensor relies on precise angular interrogation, it is acknowledged that material damping affects the sharpness of the band edge. Specifically, an increase in the loss factor was found to degrade the differential sensitivity, which decreased from roughly 30°/RIU to 15°/RIU in higher loss scenarios, which, in turn, reduced the effective Signal-to-Noise Ratio (SNR) under realistic conditions. However, the differential detection strategy mitigated this effect by canceling common-mode noise, ensuring that the sensor remained functional and accurate, even when material losses were fully considered. As shown in Figure 14, in addition to material properties, the structural robustness against fabrication tolerances was quantitatively evaluated. A Monte Carlo analysis was implemented by introducing random thickness variations of ±5% to the constituent layers over 50 independent simulation cycles. The statistical results demonstrate that the device retained a high mean differential sensitivity of 30.55°/RIU with a standard deviation of 5.31°/RIU. Although minor fluctuations in the response slope were observed due to these geometric deviations, the characteristic linearity and high sensitivity were predominantly preserved, confirming that the sensor performance remains robust and reliable under realistic manufacturing conditions.

To further highlight the practical superiority of the proposed MO-DPCS, Table 2 presents a comprehensive comparison with recently reported optical refractive index gas sensors. Fundamentally distinct from traditional passive sensors or angular SPR-based designs that rely on the absolute shift of a single resonance mode and are inherently restricted by the trade-off between sensitivity and dynamic range, our approach exploits the non-reciprocal magneto-optical effect to induce a unique “reverse-motion” of orthogonal modes. This active differential mechanism not only self-cancels common-mode interference but also constructs a robust sensing channel that allows for high-precision detection across a much wider span. Consequently, as evidenced in the table, the differential sensing architecture developed in this study provides an exceptionally broad detection range spanning from 1.000 to 1.100 RIU. Coupled with the ultra-low *LOD* established in the preceding analysis, this expansive range empowers the proposed sensor to effectively monitor a highly diverse array of critical industrial and hazardous gases.

### 3.4. Temperature Cross-Sensitivity and Environmental Stability Analysis

A primary challenge in the implementation of semiconductor-based photonic sensors is the inherent heat sensitivity of the materials involved. The permittivity of InSb is very responsive to temperature fluctuations, resulting in a refractive index alteration that may be indistinguishable from the signal produced by the target analyte. This effect, termed temperature cross-sensitivity, significantly undermines detection accuracy in real settings where thermal fluctuations are unavoidable, potentially resulting in false positive results. To evaluate the robustness of the proposed architecture against thermal noise, the spectral response was systematically characterized across a temperature range of 274 K to 275 K.

**Figure 15:**
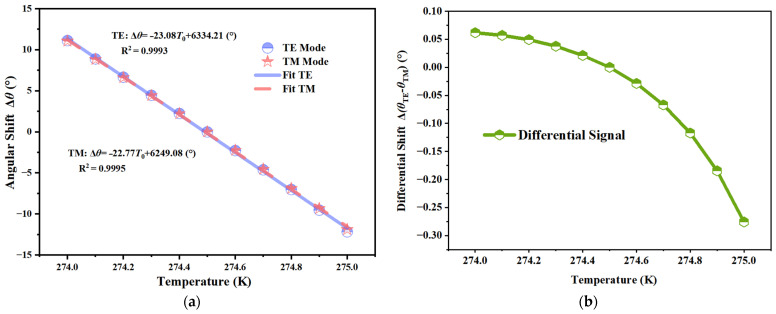
Temperature cross-sensitivity analysis of the MO-DPCS over the range 274 K to 275 K. (**a**) Angular drift of individual TE and TM modes, showing a substantial synchronous shift exceeding 20° due to the high thermal dependence of the InSb permittivity. (**b**) Variation of the differential signal under identical thermal conditions, revealing that the differential scheme effectively mitigates common-mode noise and restricts the drift to approximately 0.35°, demonstrating superior environmental stability.

Figure 15a illustrates the inherent limitations of traditional single-mode detection. With a temperature rise of just 1 K, the cutoff angles for both TE and TM modes demonstrate a significant synchronous shift, transitioning from roughly +11° to −12°. The significant angular deviation over 20° suggests that even little thermal fluctuations may be misconstrued as considerable alterations in the refractive index of the target gas, thus compromising the reliability of single-mode measurements without stringent temperature stabilizing measures.

In contrast, the differential detection strategy effectively mitigates this issue by exploiting the common-mode nature of thermal response. Since temperature-induced variations manifest as nearly identical kinematic shifts in both orthogonal polarization modes, the subtraction of the two cutoff angles inherently cancels out the majority of the thermal drift. The stability of the differential signal is presented in Figure 15b. It can be observed that the magnitude of the differential angular shift was confined within a narrow range of approximately 0.35° over the same temperature interval. Compared to the single-mode drift, the temperature cross-sensitivity was suppressed by nearly two orders of magnitude. This result confirms that the proposed differential sensor possesses a self-referencing capability, allowing it to distinguish the true refractive index signal from thermal noise and ensuring high measurement reliability in complex experimental conditions.

## 4. Conclusions

The results presented in this study demonstrate that the integration of the magneto-optical effect with 1D PCs provides a robust mechanism for enhancing refractive index sensing performance, validating the working hypothesis that the non-reciprocal nature of magnetized InSb can be manipulated to induce opposing spectral shifts in orthogonal polarization modes. Crucially, the structural efficacy was maximized through the implementation of the MODA, which enabled the precise inverse design of layer thicknesses to achieve an optimal *RC* that manual tuning could not easily attain. While previous studies have predominantly focused on simple geometric optimization to narrow resonance linewidths, the approach utilized here synergizes algorithmic design with the external magnetic field as a new degree of freedom, enabling a transition from absolute angular detection to a differential scheme that amplifies sensitivity from a negligible 0.8°/RIU to a substantial 30.8°/RIU. Interpreting these findings in a broader context, the proposed MO-DPCS addresses the critical challenge of environmental instability inherent to semiconductor-based sensing. Specifically, the differential strategy effectively converts the high thermal cross-sensitivity of InSb into a common-mode noise factor that is mathematically canceled out, thereby ensuring high measurement reliability. Future research directions should focus on the experimental realization of the proposed structure via precision growth techniques such as molecular beam epitaxy, potentially extending the concept to intelligent sensing systems where machine learning algorithms could further enhance real-time data demodulation and pattern recognition in miniaturized on-chip sensor arrays.

## Figures and Tables

**Table 2:** The proposed MO-DPCS for gas refractive index sensing compared with related research.

Refs.	Detection Range	Gas Types
[25]	RI: 1.00026~1.00046 (RIU)	N_2_O, CO_2_
[71]	RI: 1.00~1.08 (RIU)	SO*_x_*, NO*_x_*, CO
[72]	RI: 1.000265~1.000407 (RIU)	Air, N_2_, He, CO_2_
Our work	RI: 1.000~1.100 (RIU)	CH_4_ (*n* = 1.000444) CO (*n* = 1.000297) SO_2_ (*n* = 1.000683) NO_x_ (*n* ≈ 1.0002~1.0008) Air (*n* = 1.000293) Industrial volatile gases (VOCs): heavy chemical vapors (*n* ≈ 1.010~1.100)

**Table 1:** MODA optimization parameters.

Category	Parameter	Description	Value/Range
I. Global Algorithmic Configurations	*N*	Population size	30
	*M*_max_	Maximum iterations	50
	*N*_archive_	Maximum capacity of Pareto archive	100
II. Dynamic Swarming Dynamics	*r*	Dynamic neighborhood radius	Non-linear expansion: 0.25Δ → 2.25Δ
	*s*, *a*, *c*	Separation, alignment, and cohesion factors	Adaptive decay proportional to 1/*M*
	*f*, *e*	Food attraction and enemy distraction	Stochastic and iteration dependence
III. Physical Boundaries and Objective Functions	*B*_search_	Search space for external magnetic field	0.001~0.0035 (T)
	*θ*_scan_	Angular interrogation range and resolution	−90°~90°
	*Obj*_1_	Primary objective function	Min{-(*Count*_TE_ − *Count*_TM_)}
	*Obj*_2_	Secondary objective function	Min{-*Count*_TE_}

## Data Availability

Dataset available upon request from the authors.

## Supplementary Materials

The following supporting information can be downloaded at https://www.mdpi.com/article/10.3390/s26061914/s1: Figure S1: Schematic representation of a standard physical vapor deposition (PVD) system; Figure S2: The schematic diagram of the manufacturing process for the proposed layered structure. The bottom of the process flow clearly indicates the specific materials used.
