# Supplementary material for: High-Sensitivity and Temperature-Robust Gas Sensor Based on Magnetically Induced Differential Mode Splitting in InSb Photonic Crystals
Source: Sensors (Basel). 2026 Mar 18;26(6):1914. doi: 10.3390/s26061914 (PMC13030384; doi:10.3390/s26061914)
Supplement: Supplementary file 1 [file sensors-26-01914-s001.zip › sensors-4190692-supplementary.pdf]

Supplementary Materials

High-Sensitivity and Temperature-Robust Gas Sensor Based on Magnetically Induced Differential Mode Splitting in InSb Photonic Crystals

Jin Zhang [1], Leyu Chen [1], Chenxi Xu [2] and Hai-Feng Zhang [1,2,*]

[1] College of Electronic Engineering, Tongda College of Nanjing University of Posts and Telecommunications, Yangzhou 225127, China; b23020001@njupt.edu.cn
[2] College of Electronic and Optical Engineering & College of Flexible Electronics (Future Technology), Nanjing University of Posts and Telecommunications, Nanjing 210023, China
* Correspondence: hanlor@163.com or hanlor@njupt.edu.cn

S1. Physical vapor deposition (PVD)

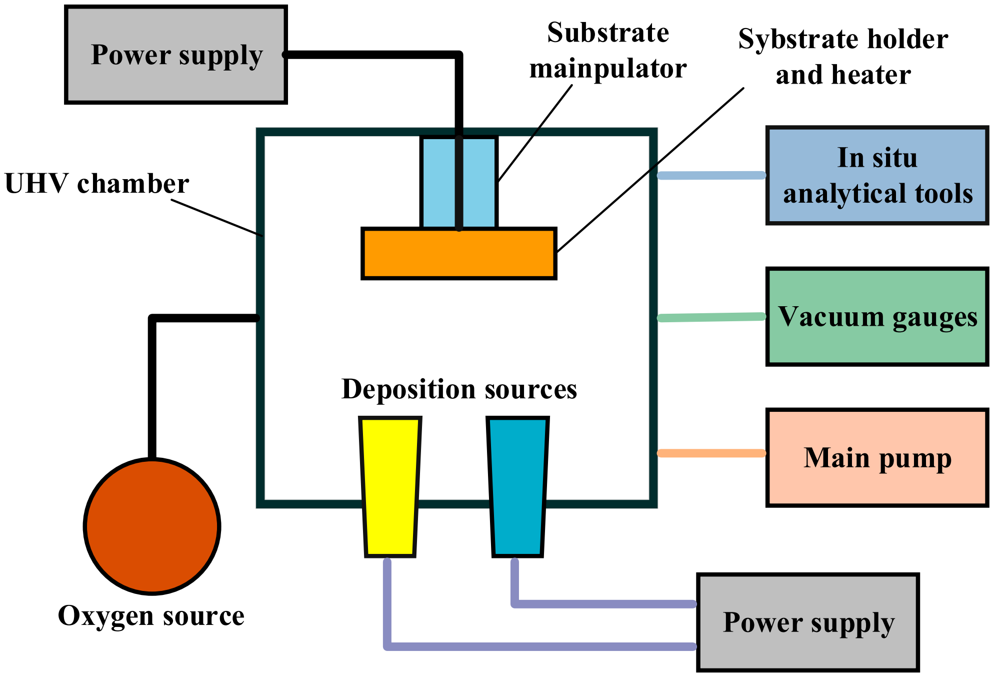

Figure S1. Schematic representation of a standard physical vapor deposition (PVD) system [2].

Physical vapor deposition (PVD) serves as a widely utilized technique for fabricating thin films, offering precise control over both layer thickness and optical characteristics [1,2]. A schematic representation of a basic PVD system is provided in Fig. S1. The procedure comprises three fundamental stages:

(i) Material vaporization: The target substance undergoes conversion into vapor through thermal excitation, ion bombardment, or laser-induced ablation.

(ii) Vapor transport: The generated gaseous species migrate across a vacuum or low-pressure atmosphere.

(iii) Film formation: Upon reaching the substrate, the vapor phase condenses, producing a uniform and strongly bonded coating.

S2. Fabrication process of the multilayered photonic structure

To realize the proposed layered configuration, a wet etching-based microfabrication approach was adopted, allowing precise control over geometry and material placement. A silicon wafer served as the primary substrate, where vertical grooves were etched and subsequently filled with different functional materials according to the designed model. The complete fabrication process is outlined below in sequential steps [3,4]:

Step 1. A silicon wafer is selected as the foundational platform due to its compatibility with standard microfabrication techniques and excellent mechanical stability.

Step 2. Vertical trenches of varying depths are patterned onto the wafer using a wet anisotropic etching technique. The groove depth is precisely adjusted to meet the dimensional requirements dictated by the scale ratio of the two integrated materials in the structural design.

Step 3. Anisotropic etching is performed in a 44 wt% potassium hydroxide aqueous solution, maintained at 85 °C, which enables directional etching with high selectivity.

Step 4. A thermally grown silicon dioxide (SiO_2) layer is employed as a hard mask, serving to protect areas outside the designated etching regions and ensure pattern fidelity.

Step 5. Once the etched trenches conform to the desired geometrical parameters, the corresponding materials are introduced into their respective grooves, completing the multilayered stacking process.

Step 6. From a theoretical perspective, when the lateral and vertical dimensions of the substrate are assumed to be unbounded, the constructed structure closely resembles the ideal model analyzed in the simulation.

The detailed procedural steps are visually summarized in Fig.S2(a)-(f).

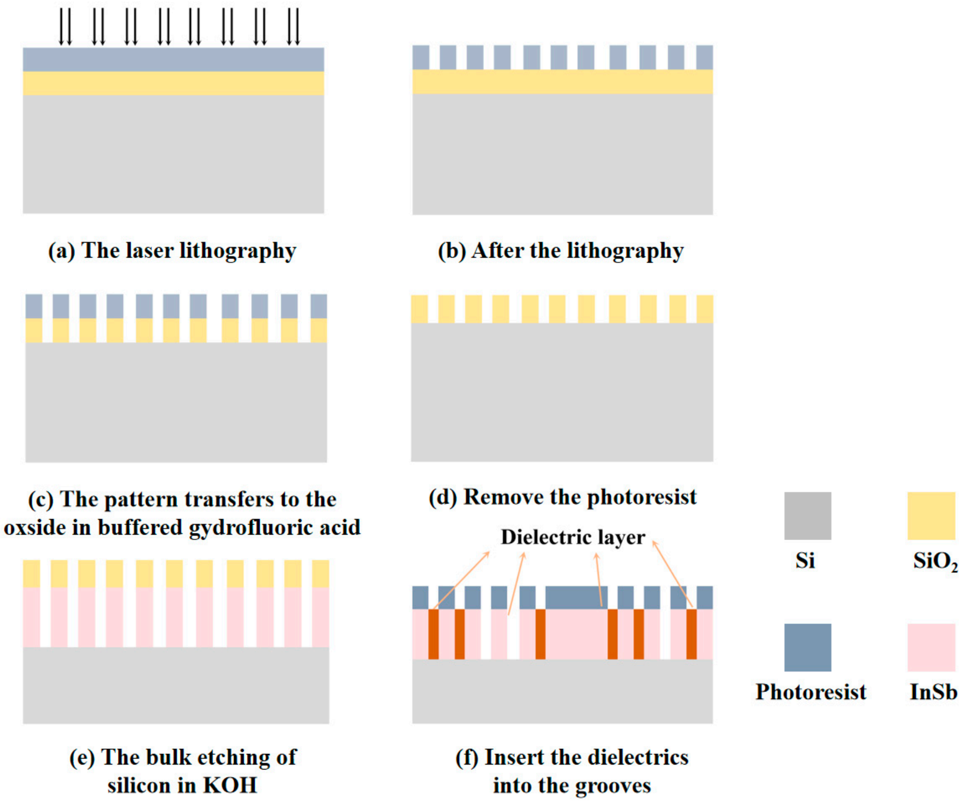

Figure S2. Schematic diagram of the manufacturing process for the proposed layered structure. The bottom of the process flow clearly indicates the specific materials used.

References

1. Wilbrandt, S.; Stenzel, O.; Kaiser, N. Experimental determination of the refractive index profile of rugate filters based on in situ measurements of transmission spectra. J. Phys. D: Appl. Phys. **2007**, 40, 1435. https://doi.org/10.1088/0022-3727/40/5/019
2. Rabe, K.M.; Ahn, C.H.; Triscone, J.M. Physics of Ferroelectrics: A Modern Perspective. 2007: Physics of Ferroelectrics: A Modern Perspective.
3. Tolmachev, V.A.; Granitsyna, L.S.; Vlasova, E.N.; Volchek, B.Z.; Nashchekin, A.V.; Remenyuk,A.D.; Astrova, E.V. One-dimensional photonic crystal obtained by vertical anisotropic etching of silicon. Semiconductors **2002**, 36, 932-935. https://doi.org/10.1134/1.1500475
4. Guo, S.J.; Hu, C.X.; Zhang, H.F. Ultra-wide unidirectional infrared absorber based on 1D gyromagnetic photonic crystals concatenated with general Fibonacci quasi-periodic structure in transverse magnetization. J. Opt. **2020**, 22, 105101. https://doi.org/10.1088/2040-8986/abad09
